# MT5-MMP promotes neuroinflammation, neuronal excitability and Aβ production in primary neuron/astrocyte cultures from the 5xFAD mouse model of Alzheimer’s disease

**DOI:** 10.1186/s12974-022-02407-z

**Published:** 2022-03-11

**Authors:** Dominika Pilat, Jean-Michel Paumier, Laura García-González, Laurence Louis, Delphine Stephan, Christine Manrique, Michel Khrestchatisky, Eric Di Pasquale, Kévin Baranger, Santiago Rivera

**Affiliations:** 1 Aix-Marseille Univ, CNRS, INP, Inst Neurophysiopathol, Marseille, France; 2 Present Address: Department of Neurology, Northwestern University Feinberg School of Medicine, Chicago, IL USA; 3 Present Address: BarcelonaBeta Brain Research Center (BBRC), Pasqual Maragall Foundation, Barcelona, Spain

**Keywords:** Neuroinflammation, IL-1β, Amyloid peptide, Amyloid precursor protein, C99, Synaptic activity, Matrix metalloproteinase, Neuroprotection, AAV, Patch-clamp

## Abstract

**Background:**

Membrane-type matrix metalloproteinase 5 (MT5-MMP) deficiency in the 5xFAD mouse model of Alzheimer's disease (AD) reduces brain neuroinflammation and amyloidosis, and prevents deficits in synaptic activity and cognition in prodromal stages of the disease. In addition, MT5-MMP deficiency prevents interleukin-1 beta (IL-1β)-mediated inflammation in the peripheral nervous system. In this context, we hypothesized that the MT5-MMP/IL-1β tandem could regulate nascent AD pathogenic events in developing neural cells shortly after the onset of transgene activation.

**Methods:**

To test this hypothesis, we used 11–14 day in vitro primary cortical cultures from wild type, MT5-MMP^−/−^, 5xFAD and 5xFAD/MT5-MMP^−/−^ mice, and evaluated the impact of MT5-MMP deficiency and IL-1β treatment for 24 h, by performing whole cell patch-clamp recordings, RT-qPCR, western blot, gel zymography, ELISA, immunocytochemistry and adeno-associated virus (AAV)-mediated transduction.

**Results:**

5xFAD cells showed higher levels of MT5-MMP than wild type, concomitant with higher basal levels of inflammatory mediators. Moreover, MT5-MMP-deficient cultures had strong decrease of the inflammatory response to IL-1β, as well as decreased stability of recombinant IL-1β. The levels of amyloid beta peptide (Aβ) were similar in 5xFAD and wild-type cultures, and IL-1β treatment did not affect Aβ levels. Instead, the absence of MT5-MMP significantly reduced Aβ by more than 40% while sparing APP metabolism, suggesting altogether no functional crosstalk between IL-1β and APP/Aβ, as well as independent control of their levels by MT5-MMP. The lack of MT5-MMP strongly downregulated the AAV-induced neuronal accumulation of the C-terminal APP fragment, C99, and subsequently that of Aβ. Finally, MT5-MMP deficiency prevented basal hyperexcitability observed in 5xFAD neurons, but not hyperexcitability induced by IL-1β treatment.

**Conclusions:**

Neuroinflammation and hyperexcitability precede Aβ accumulation in developing neural cells with nascent expression of AD transgenes. MT5-MMP deletion is able to tune down basal neuronal inflammation and hyperexcitability, as well as APP/Aβ metabolism. In addition, MT5-MMP deficiency prevents IL-1β-mediated effects in brain cells, except hyperexcitability. Overall, this work reinforces the idea that MT5-MMP is at the crossroads of pathogenic AD pathways that are already incipiently activated in developing neural cells, and that targeting MT5-MMP opens interesting therapeutic prospects.

**Supplementary Information:**

The online version contains supplementary material available at 10.1186/s12974-022-02407-z.

## Background

Membrane-type matrix metalloproteinase 5 (MT5-MMP; also known as MMP-24) is a member of the matrix metalloproteinase (MMP) family of Zn^2+^-dependent pleiotropic endopeptidases [1]. MT5-MMP is the only MMP preferentially expressed in the nervous system [2] and is involved in different forms of neural cell plasticity (reviewed in [3, 4]) that include axonal outgrowth [5], post-lesion axonal sprouting [6], and neural stem cell differentiation of precursor cells expressing glial fibrillary acidic protein (GFAP) [7]. Only a handful of MT5-MMP interacting proteins or substrates have been identified, providing potential clues for the interpretation of MT5-MMP functions. Thus, MT5-MMP interacts with the AMPA receptor binding protein (ABP) and the glutamate receptor-interacting protein (GRIP) [8], both hosting PDZ domains that drive AMPA receptor targeting to the plasma membrane. MT5-MMP also cleaves E- and N-cadherins [7–10], involved in synapse organization and stability. Amyloid precursor protein (APP) is another relevant substrate of MT5-MMP [11–13], which holds a central position in Alzheimer’s disease (AD) pathogenesis. APP processing by α- and β-secretase generates C-terminal fragments (CTF) known as C83 and C99, respectively. Further intramembrane processing of C99 by γ-secretase releases the amyloid beta peptide (Aβ). Accumulation of C99 and Aβ is a hallmark of the pathogenic amyloidogenic cascade in Alzheimer’s disease (AD) [14], while C83 is the major physiological APP fragment generated by human [15] and murine [16] neurons. Alternatively to canonical APP processing, MT5-MMP has been shown to cleave APP and to generate an η-CTF from which subsequent processing by α-secretase releases an Aη-α fragment that inhibits LTP in cellulo [12]. We found this cleavage to occur in vivo in the brains of the 5xFAD mouse model of AD [13]. MT5-MMP is a new pro-amyloidogenic factor, whose deficiency markedly reduced the levels of Aβ and C99 in early stages of the pathology, concomitant with prevention of deficits in long-term potentiation (LTP), spatial learning and working memory [13, 17]. MT5-MMP deficiency also prevented glial reactivity and the increase in the levels of pro-inflammatory interleukin-1 beta (IL-1β) in 5xFAD mice [13]. IL-1β is a major neuroinflammatory mediator, highly expressed in AD following activation of the NLRP3 inflammasome [18–20], and shows complex and divers effects on neurons including disruption of synaptic plasticity [21], promotion of excitotoxicity [22, 23] and α- and γ-secretase activities, while reducing β-secretase activity [24, 25] and Aβ levels [26, 27]. Interestingly, IL-1β failed to induce the expected neuroinflammation after injection into the paws of MT5-MMP-deficient mice in a model of thermal pain, unveiling functional interactions between MT5-MMP and IL-1β in the peripheral nervous system (PNS) through a mechanism involving N-cadherin [10].

Together, these data extend the scope of MT5-MMP actions beyond APP processing, as previously suggested [28] and led us to hypothesize that MT5-MMP modulates, possibly in concert with IL-1β, three major events in AD: APP/Aβ metabolism, neuroinflammation, and neuronal activity. It was also our objective to explore the possibility that such modulations occur in young neural cells of 5xFAD brains, well before the first pathological signs. We tested these hypotheses using mixed neuron/astrocyte primary cortical cultures from wild type (WT), 5xFAD (Tg), MT5-MMP^−/−^ (MT5^−/−^) and 5xFAD/MT5-MMP^−/−^ (TgMT5^−/−^) mice [13, 17] stimulated or not by IL-1β. Our study reveals that MT5-MMP modulates Aβ and C99, IL-1β-mediated inflammation, and synaptic activity in young neurons, overall highlighting a key role for this proteinase in early molecular and cellular events that may preconfigure AD pathology.

## Materials and methods

### Mixed neuronal glial cultures and treatments

To generate mixed neuronal–glial cell cultures, we used WT, MT5^−/−^, Tg and TgMT5^−/−^ mice in a C57BL6 genetic background as previously described [13, 17]. All the experimental procedures were conducted in agreement with the authorization for animal experimentation attributed by the French Ministry of Research to the laboratory (research project: APAFIS#23040-2019112708474721 v4). Briefly, pregnant females were deeply anesthetized with xylazine (15 mg/kg) and ketamine (150 mg/kg) (Ceva Santé animale, Libourne, France), and E16 embryos were extracted from the uterine horns and cerebral cortices dissected. All the culture media, fetal bovine serum (FBS), reagents and supplements for cell culture were purchased from ThermoFisher Scientific (Villebon-sur-Yvette, France). Cortices were placed into cold HBSS1X medium and dissociated for 10 min at 37 °C in HBSS1X containing DNAse I (10 µg/mL) and 0.1% trypsin. Reaction was stopped by the addition of a DMEM solution containing 10% FBS and further mechanical dissociation was performed through a pipette cone. After centrifugation for 5 min at 300×*g*, 3.10^5^ cells/well were plated onto 6-well plates pre-coated with poly-l-lysine (10 μg/mL, Sigma-Aldrich, Saint-Quentin Fallavier, France) for 2 h in DMEM medium containing 10% FBS and 1% penicillin/streptomycin (P/S). This medium was further replaced by Neurobasal containing B27, 1% glutamine and 1% P/S for 11 days in vitro (DIV) without anti-mitotic agent. Cells were treated or not with IL-1β (10 ng/mL, PeproTech, Neuilly-sur-Seine, France) and/or DAPT (10 μM, Tocris, Bio-Techne, Lille, France) or proteasome inhibitor MG132 (5 μM, Enzo Life Science, Lyon, France), 24 h before being collected  in either RIPA buffer (Sigma-Aldrich) for western blot (WB) analysis or collected for RNA extraction. For immunocytochemistry (ICC) experiments, cells were plated at 1.10^5^ density on 24-well plates on coverslips pre-coated with 500 μg/mL of poly-l-lysine. For electrophysiological experiments, cells were plated as described above for ICC and recorded between 11 and 14 DIV.

### MTT test

Cell viability was evaluated using the 3-(4,5-dimethylthiazol-2yl)-2,5-diphenyl tetrazolium bromide (MTT) assay (Sigma-Aldrich), which measures mitochondrial activity in living cells. A solution at 5 mg/mL was prepared into Neurobasal and then added to cultures at a final concentration of 0.5 mg/mL for 3 h at 37°C, 5% CO_2_. Media were fully removed and 200 μL of DMSO added, then 100 μL of DMSO were transferred into a 96-well plate and absorbance (OD) at 550 nm was read in a spectrophotometer. Data were calculated as the percentage of living cells = (transfected cell OD_550_/control cell OD_550_) × 100. The mean values ± SEM were obtained from at least five animals by genotype.

### Viral infections

An empty AAV10 or encoding human C99 under control of the synapsin-1 promoter (AAV-empty or AAV-C99 thereafter) were previously described [29]. WT and MT5^−/−^ neurons were transduced at 6 DIV with 2 μL (at 5.10^12^ vg/mL, MOI = 2.5 × 10^4^), treated at 10 DIV with DAPT (10 μM) and recovered at 11 DIV for WB analyses.

### Western blot

Protein concentration was determined using a Bio-Rad *DC*™ protein assay kit (Bio-Rad, Marnes-La-Coquette, France). Proteins (30 μg) were loaded and run on 10–15% SDS-PAGE gels, or 4–20% Tris–Glycine pre-casted gels or low molecular weight 16% Tris–Tricine pre-cast gels (ThermoFisher Scientific) and transferred onto nitrocellulose membranes (Dutscher, Brumath, France). After blocking, membranes were probed with the following antibodies directed against MT5-MMP (1/500, our own antibody previously described [13]), or APP N-terminal fragment (22C11, 1/1000, Millipore, Merck Millipore, Molsheim, France), APP C-terminal fragment (APP-CTF, 1/1000, Sigma-Aldrich), human Aβ (6E10, 1/500, Ozyme, Saint-Cyr l’Ecole, France), Aβ/C99 (82E1, 1/100, IBL America, Illkirch-Graffenstaden, France), GFAP (1/1000, Millipore), IL-1β (1/500, PeproTech), N-cadherin (1/1000, BD Biosciences, Le Pont de Claix, France), MAP-2 (1/500, Sigma-Aldrich), β-III tubulin (1/1000, Sigma-Aldrich), LRP-1 (1/1000, Abcam, Cambridge, United Kingdom), RAGE (1/1000, Abcam), LDLR (1/1000, Proteintech Europe, Manchester, United Kingdom), LRP-8 (1/250, Abcam), Histone 3 (1/1000, Abcam), Na^+^/K^+^ ATPase (1/1000, Abcam), β-actin (1/5000, Sigma-Aldrich), GAPDH (1/5000, Sigma-Aldrich), and then incubated with horseradish peroxidase-conjugated secondary IgG antibodies (Jackson Immunoresearch, Interchim, Montluçon, France). Note that depending on the molecular weight of the proteins studied and thus the gels used, GAPDH, β-actin or ponceau S staining of the membrane [30, 31] were used as loading and normalization controls. Immunoblot signals were visualized using the ECL chemiluminescence kit (Dutscher) and quantified using Fiji/Image J software (NIH). Note that immunoblots were represented in separated columns when bands from the same membrane were not adjacent.

### Subcellular fractionation

Cytoplasmic, membranous, and nuclear fractions were prepared from cell lysates using a ProteoExtract^®^ Subcellular Proteome Extraction Kit (Calbiochem, Merck Millipore, Molsheim, France) according to the manufacturer’s instructions. The purity of each fraction was analyzed by western blot as described above, using antibodies against GAPDH, Na^+^/K^+^ ATPase and Histone 3 for cytoplasmic, membranous and nuclear fractions, respectively.

### Gel zymography

We used gelatin zymography on culture supernatants to assess changes in the levels of MMP-2 and MMP-9, also known as gelatinase A and B, respectively. As previously described [32], equal amounts of serum-free supernatants in non-denaturing and non-reducing conditions were subjected to zymography according to the manufacturer’s recommendations (ThermoFisher Scientific). Gels were scanned using GeneTools software.

### Reverse transcription-quantitative polymerase chain reaction (RT-qPCR)

Total RNA was extracted from 11 DIV cells using the Nucleospin RNA kit (Macherey–Nagel, Hoerdt, France) according to the manufacturer’s recommendations. All the reagents for RT-qPCR experiments were purchased from ThermoFisher Scientific. Single-stranded cDNA was synthesized from 500 ng of RNA using the High-Capacity RNA to cDNA™ kit adapted for quantitative PCR. Twenty-five ng of cDNA were subjected to a qPCR reaction using the Fast Real-Time PCR System. For each experiment, cDNA samples were analyzed in duplicate and relative gene expression was obtained using the comparative 2^−(ΔΔCt)^ method after normalization to the *Gapdh* (Mm99999915_g1) housekeeping gene [32, 33]. The expression of the following genes was measured: *Mmp24* (Mm00487721_m1), *Mmp14* (Mm00485054_m1), *Il-1*β (Mm01336189_m1), *Gfap* (Mm01253033_m1), *Ccl2* (Mm00441242_m1), *Mmp2* (Mm00439498_m1), *Mmp9* (Mm00442991_m1), *Ide* (Mm00473077_m1), *Ace* (Mm00802048_m1), *Ece* (Mm01187091_m1), *Tnfa* (Mm00443258_m1), *Bace1* (Mm00478664_m1), *Psen1* (Mm00501184_m1), *Adam10* (Mm00545742_m1), *Lrp1* (Mm0046458_m1), *Lrp8* (Mm00474023_m1), *Ldlr* (Mm00440169_m1), *Ager* (Mm00545815_m1), *APP* (Hs00169098_m1), *PSEN1* (Hs00997789_m1).

### Immunocytochemistry

After 11 DIV, our neural cultures were fixed for 15 min with AntigenFix (Diapath, MM France, Brignais, France) and blocked with PBS1X, BSA 3%, 0.1% Triton X-100 (blocking solution) for 1 h. Primary antibodies GFAP (Dako France, Trappes, France), Iba1 (Wako, Sobioda, Mont-Bonnot Saint-Martin, France), β-III tubulin (Sigma-Aldrich), were used at 1/500 dilution. Appropriate AlexaFluor-coupled secondary antibodies were used at 1/800 dilution. Hoechst 33342 (0.5 μg/mL) was used to stain the nuclei. Antibodies and Hoechst were diluted in blocking solution. Omission of the primary antibody was used as control and no immunostaining was observed. Samples were mounted using Prolong Gold Antifading reagent on Superfrost glass slides (Dutscher). Images were taken and processed using a confocal microscope (LSM 700) and Zen software (Zeiss, Jena, Germany).

### ELISA

Total Aβ38, Aβ40 and Aβ42 levels in culture supernatants were assessed by ELISA using the V-PLEX Plus Aβ Peptide Panel 1 (4G8) Kit (Meso Scale Discovery, Rockville, Maryland, USA) according to the manufacturer's recommendations. The MSD kit uses 4G8 as a capture antibody that recognizes both mouse and human Aβ. Specific Aβ38, Aβ40 and Aβ42 were used for detection. Therefore, the measured Aβ levels are a mixture of endogenous mouse Aβ and human Aβ derived from the processing of human APP. Human Aβ40 levels in culture supernatants after AAV-C99 infections were evaluated using the human Aβ40 ELISA kit (#KHB3481, ThermoFisher Scientific). For detection of IL-1β and MCP-1 in supernatants, we used the murine IL-1β and MCP-1 ELISA Development Kits (PeproTech). IL-6 protein levels were measured using V-PLEX Proinflammatory Panel 1 mouse Kit (K15048D-1, Meso Scale Discovery, Rockville, Maryland, USA) and analyses were done using a QuickPlex SQ 120 instrument (MSD) and DISCOVERY WORKBENCH^®^ 4.0 software. All these assays were used as recommended by the manufacturers.

### Electrophysiology

#### Patch-clamp

Whole-cell recordings were performed on neurons with pyramidal shape using an Axopatch200B amplifier (Axon Instruments, Axon Digidata 1550, Molecular Device, San José, California) under visual control, using a Zeiss Examiner A1 infraRed differential interference contrast microscope (Zeiss Meditech, Marly le Roi, France) coupled to a Jenoptik ProgRes MF camera (Carl Zeiss, Jena, Germany). Patch microelectrodes (1.5 mm OD, borosilicate filament glass, BF150 from WPI) were pulled using a PP-830 electrode puller (Narishige, Fulbourn, Cambridge, UK), filled with 100 mM CsCl, 30 mM CsFl, 10 mM N-2-hydroxyethylpiperazine-N-2-ethanesulphonic acid (HEPES), 5 mM ethylene glycol-bis (b-aminoethylether)-N,N,N’, N-tetraacetic acid (EGTA), and 1 mM MgCl_2_. Two mM CaCl_2_ and 4 mM Mg-ATP/0.4 mM Na_2_-GTP was added on the day of the experiment (pH 7.4, balanced with CsOH). Pipettes (4–6 MΩ) were directed onto neurons using a motorized Sutter microdrive (ROE200, Sutter Instrument Co WPI, Friedberg, Germany). The offset between the reference electrode and the patch pipette was zeroed upon contact of the recording chamber extracellular medium (aCSF, artificial Cerebro-Spinal Fluid 140 mM NaCl, 3 mM KCl, 10 mM Hepes, 10 mM glucose, 2.5 mM CaCl_2_, 1 mM MgCl_2_, 300 nM TTX, pH 7.4 with NaOH). The reference electrode was an Ag–AgCl wire connected to the extracellular solution. Selected pyramidal neurons had gigaohm seals (typically 1–5 GΩ), a stable resting membrane potential and an access resistance < 15 MΩ that was not compensated for.

#### Recording and analysis of baseline synaptic transmission

In voltage-clamp mode, cells were held at -50 mV and miniature global post-synaptic currents (gPSCs) were recorded for 5 min (band width, 1 kHz), after a 5-min recovery from breaking through the plasma membrane. We did not make any distinction between excitatory or inhibitory synaptic currents. The analysis was run offline using the Clampfit11 (Axon Instruments) routines. gPSCs were selected individually for each neuron of each genotype and pharmacological condition. Statistics were then obtained regarding the mean amplitude and the mean frequency of gPSCs occurrence during 5 min to generate the histograms.

### Statistics

All values represent the means ± SEM of the number of independent cultures indicated in the figure legends. For statistical analyses, we used ANOVA followed by a Fisher’s LSD post hoc test and set the statistical significance at *p* < 0.05. Analyses were performed with the GraphPad Prism software (San Diego, California USA).

## Results

### Neural 5xFAD cells show upregulation of MT5-MMP, but its deficiency and IL-1β treatment do not impact cell stability

**Fig. 1:**
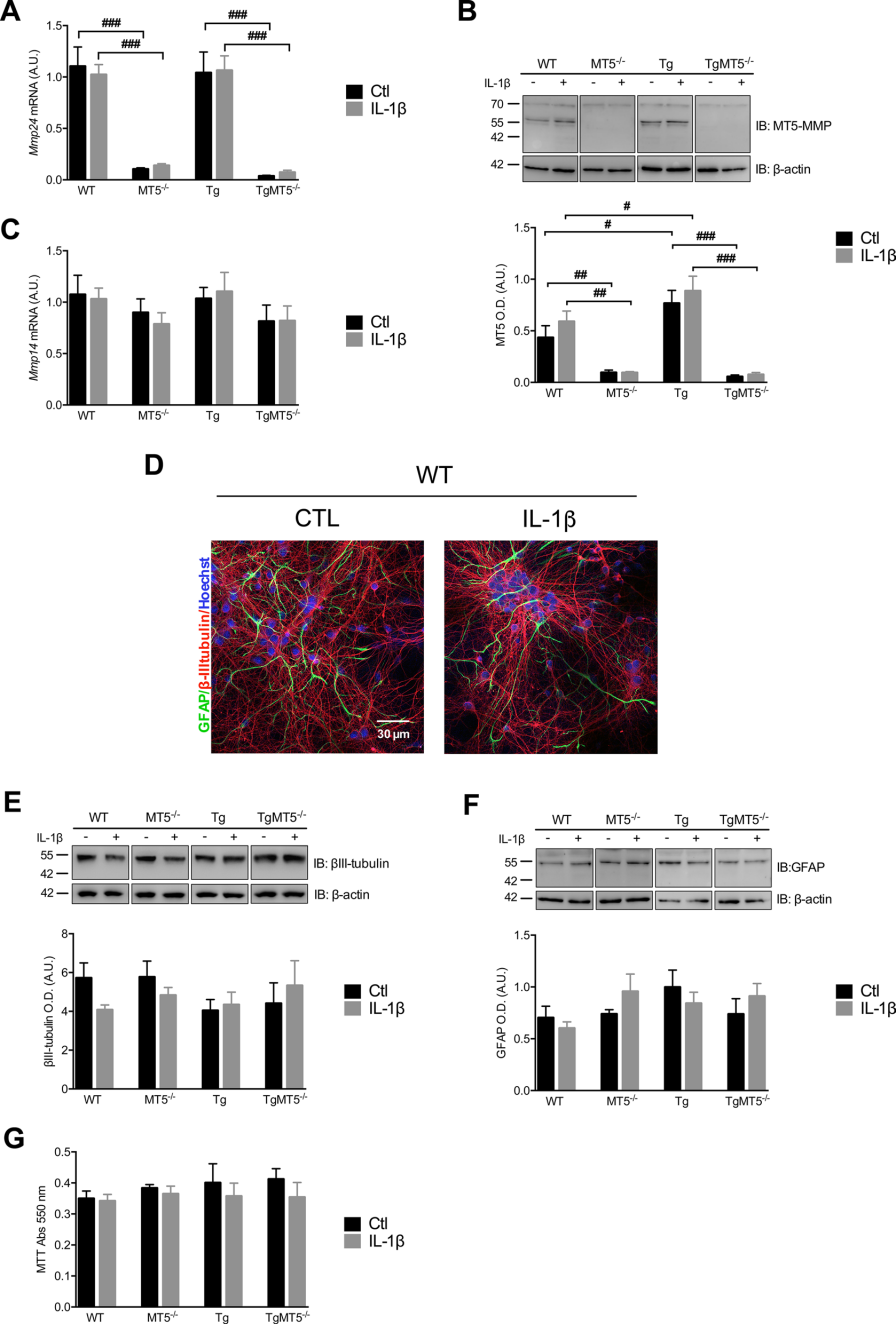
Effects of MT5-MMP deficiency and IL-1β treatment on primary cultures of cortical neural cells.** A** mRNA levels of *Mmp24* analyzed by RT-qPCR. Data values were normalized by *Gapdh* as housekeeping gene. **B** MT5-MMP levels detected by immunoblot (top panel) with its corresponding quantification (lower panel) normalized with β-actin. Note that MT5-MMP was not detected in MT5^−/−^ and TgMT5^−/−^ cells. **C** mRNA levels of *Mmp14* analyzed by RT-qPCR. Data values were normalized by *Gapdh* as housekeeping gene. **D** Representative confocal micrographs of primary neuronal cultures from WT mice treated or not with IL-1β and labeled with astrocytic marker GFAP (green) and neuronal marker β-III tubulin (red). Nuclei are stained with Hoechst (blue). Scale bar: 30 μm. **E** and **F** Detection of β-III tubulin and GFAP levels by immunoblots (top panels) with their corresponding quantifications (lower panel) normalized with β-actin. **G** Histogram showing the quantification of cell viability using the MTT assay. **A**–**C** and **E**–**G**, Black bars represent control (untreated) conditions and grey bars IL-1β treated conditions (10 ng/mL for 24 h). Values for **A**–**C** are the mean ± SEM of 6–8 independent cultures by genotype, for **E** and **F** of 4 independent cultures by genotype and for **G** of 3-7 independent cultures by genotype. Values are presented as % of the control. ^#^*p* < 0.05, ^##^*p* < 0.01 and ^###^*p* < 0.001 between genotypes. ANOVA followed by post hoc Fisher’s LSD test. *IB* Immunoblot, *O.D*. optical density, *A.U.* arbitrary units

As expected, neuron/astrocyte cultures from MT5^−/−^ mice did not express *Mmp24* mRNA, which encodes MT5-MMP (Fig. 1A). In addition, there was no difference in *Mmp24* mRNA levels between WT and Tg cells, even after IL-1β treatment (10 ng/mL) (Fig. 1A). We confirmed the depletion of MT5-MMP protein in our cells (Fig. 1B) and also found a 76% increase of MT5-MMP levels in Tg cells compared to WT that was maintained under IL-1β (Fig. 1B). *Mmp14* (MT1-MMP) is a close homolog of *Mmp24* (MT5-MMP) which shares pro-amyloidogenic features [33, 34]. Accordingly, we looked for possible compensatory regulations*,* but *Mmp14* mRNA remained stable in all genotypes and thus did not compensate for MT5-MMP deficiency, regardless of IL-1β treatment (Fig. 1C).

Cultures were roughly estimated at 2/3 of neurons (β-III tubulin^+^) and 1/3 of astrocytes (GFAP^+^), while no microglia was detected (Iba1^+^). A representative image of WT neural cells, treated or not with IL-1β, is shown in Fig. 1D. Moreover, levels of the β-III tubulin (Fig. 1E) and MAP-2 (not shown) neuronal markers were stable across genotypes in all experimental conditions, as revealed by WB. Likewise, there was no change in the content of the astrocytic marker GFAP (Fig. 1F). Moreover, the MTT test confirmed no cytotoxic effects associated with genotypes or IL-1β treatment in our conditions (Fig. 1G).

### The expression of genes coding for inflammatory mediators is selectively altered in MT5-MMP-deficient cells

**Fig. 2:**
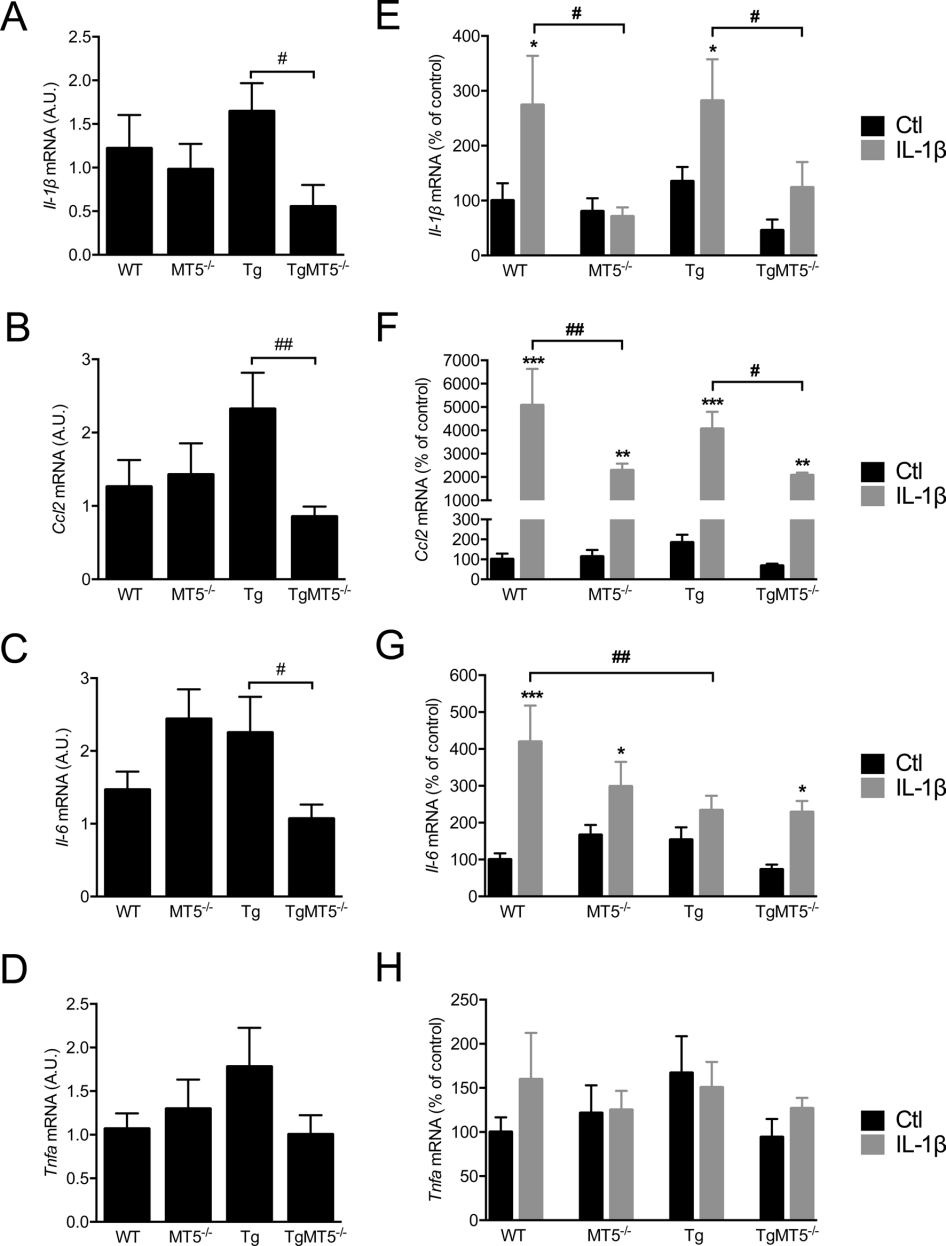
Effects of MT5-MMP deficiency on IL-1β-mediated neuroinflammation in cortical neural cell cultures. **A**–**D** Analyses of *Il-1*β, *Ccl2*, *Il-6* and *Tnfa* basal mRNA expression in primary neural cells by RT-qPCR and normalized by *Gapdh* as housekeeping gene. Note the consistent significant decrease of *Il-1*β, *Ccl2* and *Il-6* mRNA levels in TgMT5^−/−^ cells compared to Tg. **E–H** Analyses of *Il-1*β, *Ccl2*, *Il-6* and *Tnfa* mRNA expression in primary neural cells by RT-qPCR and normalized by *Gapdh* as housekeeping gene. Black bars represent control (untreated) conditions and grey bars IL-1β treated conditions (10 ng/mL for 24 h). Values for **A**–**H** are the mean ± SEM of 6–10 independent cultures by genotype. *p < 0.05, and ***p < 0.001 between untreated and treated cultures in the same genotype; ^#^*p* < 0.05 and ^##^*p* < 0.01 between genotypes. ANOVA followed by post hoc Fisher’s LSD test. *A.U.* arbitrary units

As previously shown in the peripheral nervous system, MT5-MMP deficiency appeared to interfere with IL-1β-mediated response [10]. Accordingly, we questioned whether this might also be the case in the central nervous system (CNS). We first analyzed the effect of genotype on basal levels of key AD inflammatory mediators and found that IL-1β mRNA was drastically decreased by 77% in TgMT5^−/−^ cells compared with Tg (Fig. 2A). Similarly, *Ccl2* mRNA, which encodes monocyte chemoattractant protein-1 (MCP-1) (Fig. 2B) and *Il-6* mRNA were respectively significantly decreased by 63% and 52% in TgMT5^−/−^ cells, compared with Tg (Fig. 2C). Only *Tnfa* (TNF-α) expression remained unchanged between genotypes (Fig. 2D).

IL-1β is a key cytokine in AD [20] that stimulates its own expression [35, 36] as well as that of other inflammatory mediators, including *Il-6*, *Ccl2* and *Tnfa* [37–40]. In pace with these data, IL-1β induced its own mRNA in WT (174%) and Tg (109%) cultures (Fig. 2E), but not in cells lacking MT5-MMP (MT5^−/−^ and TgMT5^−/−^) (Fig. 2E). *Ccl2* mRNA levels, which were relatively low in basal conditions, respectively reached 5000% and 4000% increases in WT and Tg cells after IL-1β exposure (Fig. 2F). Again, the stimulating effect of IL-1β on *Ccl2* was hampered by MT5-MMP deficiency, with mRNA levels significantly reduced by 55% and 49% in MT5^−/−^ and TgMT5^−/−^ cells compared with their respective WT and Tg controls. IL-1β treatment upregulated *Il-6* levels by 318% in WT, 79% in MT5^−/−^ and 188% in TgMT5^−/−^ cells, but had no effect in Tg cells, whose *Il-6* levels were down by 45% compared to IL-1β-treated WT (Fig. 2G). On the contrary, unlike other neuroinflammatory mediators, *Tnfa* expression was not affected by IL-1β in the present experimental conditions (Fig. 2H). Overall, MT5-MMP deficiency modulates the expression of *Il-1β*, *Ccl2* and *Il-6* while sparing that of *Tnfa*, which is otherwise  unaffected by genotypes or 10 ng/mL IL-1β treatment.

### The levels of MCP-1 and IL-1β proteins decrease in MT5-MMP-deficient cells

**Fig. 3:**
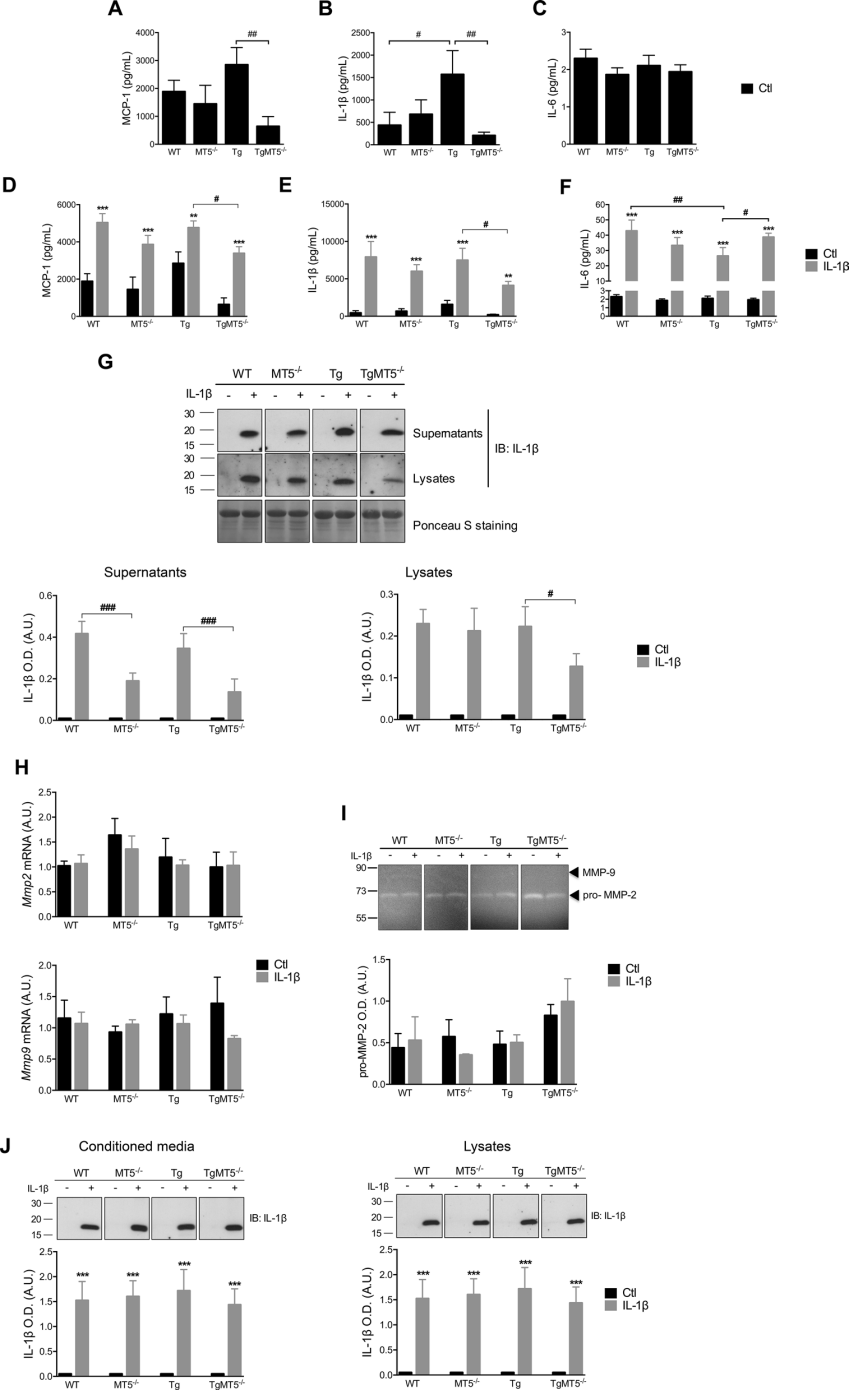
Effects of MT5-MMP deficiency on pro-inflammatory protein levels and IL-1β stability in cortical neural cells.** A**–**C** Measurement of MCP-1, IL-1β and IL-6 levels (pg/mL) in primary cultures by ELISA. Note the significant decrease of MCP-1 and IL-1β levels in TgMT5^−/−^ compared with Tg cells. **D**–**F** Measurement of MCP-1, IL-1β and IL-6 levels (pg/mL) in primary cultures by ELISA upon IL-1β treatment (10 ng/mL for 24 h). Black bars represent control (untreated) conditions and grey bars IL-1β treated conditions (10 ng/mL for 24 h). **G** Immunoblots (top panel) and the corresponding ponceau normalized quantification (lower panels) of IL-1β levels in supernatants and cell lysates after 24 h of incubation with 10 ng/mL IL-1β. Note that the levels of IL-1β were affected in the absence of MT5-MMP. **H** RT-qPCR analysis of mRNA levels of *Mmp2* (upper panel) and *Mmp9* (bottom panel) in primary cultures, normalized by *Gapdh* as housekeeping gene. **I** Zymogram (upper panel) and the corresponding quantification (bottom panel) of pro-MMP-2 levels in primary neural cultures. **J** Immunoblot analyses of IL-1β levels after incubation for 24 h at 37 °C in cell conditioned supernatants and lysates with 10 ng/mL IL-1β. Note that none of the the conditioned media modified IL-1β stability after 24 h incubation. Values for **A**–**D**, **E** and **H** are the mean ± SEM of 6–7 independent cultures by genotype and for **F** and **G** 3–6 independent cultures. Values are presented as % of the control. *p < 0.05, **p < 0.01 and ***p < 0.001 between untreated and treated cultures in the same genotype; ^#^*p* < 0.05, ^##^*p* < 0.01 and ^###^*p* < 0.001 between genotypes. ANOVA followed by post hoc Fisher’s LSD test. *IB* immunoblot, *O.D.* optical density, *A.U.* arbitrary units

Considering changes observed at the mRNA level, we used ELISA to determine whether protein levels of MCP-1, IL-1β and IL-6 were affected by genotype or IL-1β treatment. Basal MCP-1 levels were below 2000 pg/mL in WT and MT5^−/−^ cells, while reaching values close to 3000 pg/mL in Tg cells, which were not significantly different from WT (Fig. 3A). In contrast, TgMT5^−/−^ cells exhibited a statistically significant drop (~ 600 pg/mL) of 77% compared with Tg (Fig. 3A). The concentration of endogenous IL-1β was around 500 pg/mL in WT and MT5^−/−^ cells, and 259% higher in Tg cells. Such increase was prevented in TgMT5^−/−^ cells, whose values were 87% lower than Tg (Fig. 3B). IL-6 levels were comparatively very low and unchanged in all experimental conditions (Fig. 3C).

Inflammatory challenge with IL-1β strongly upregulated MCP-1 levels in all genotypes, but they remained significantly lower by 29% in TgMT5^−/−^ cultures compared with Tg (Fig. 3D). The burst in IL-1β concentration detected by ELISA in cell supernatants after treatment likely reflects the addition of recombinant cytokine, although it cannot be excluded that a small portion results from endogenous synthesis. In any case, IL-1β levels in TgMT5^−/−^ cells were significantly lower (45%) compared with Tg (Fig. 3E). IL-6 levels were also strongly upregulated by IL-1β, but in this case the major change was the 38% reduction in Tg levels compared to WT and the recovery of IL-6 levels in TgMT5^−/−^ cells to near WT values (Fig. 3F). After treatment with recombinant IL-1β, a 17 kDa immunoreactive band matching the size of the active form of the cytokine, and absent in untreated cultures, was detected by WB in cell supernatants, (Fig. 3G). The band intensity was reduced in TgMT5^−/−^ (62%) and MT5^−/−^ (56%) cells, compared with Tg and WT, respectively (Fig. 3G). The reductions in extracellular IL-1β, prompted us to assess its content in cell lysates, inferring a possible increase in cellular uptake of the cytokine and consequent intracellular accumulation. However, this was not the case, as cell lysates also revealed a 46% reduction of IL-1β levels in TgMT5^−/−^ cells compared with Tg, while no differences were observed between MT5^−/−^ and WT cells (Fig. 3G).

### Genotype-dependent degradation of IL-1β

These results raised the possibility that IL-1β may be more efficiently eliminated in the microenvironment of MT5-MMP-deficient cells. Two MT5-MMP homologs, MMP-2 and MMP-9 (also known as gelatinases A and B, respectively), have been shown to degrade IL-1β, thus likely contributing to the resolution of inflammation under certain circumstances [41]. We therefore evaluated a possible compensatory upregulation of these soluble MMPs that could explain IL-1β degradation upon MT5-MMP deficiency. However, no changes were observed in the mRNA levels encoding MMP-2 and MMP-9 (Fig. 3H). Moreover, a single band of gelatinolysis with the expected molecular weight of pro-MMP-2 appeared in highly sensitive gelatin zymograms, and this band remained stable across genotypes or after IL-1β treatment (Fig. 3I). The lower molecular weight active form of MMP-2 (~ 64 kDa) and MMP-9 (~ 90 kDa) were virtually undetectable in these conditions (Fig. 3I).

We next asked whether, more generally, proteolytic activities located in intracellular or extracellular environments could explain the putative degradation of IL-1β. To this end, we incubated recombinant IL-1β for 24 h in cell-free conditioned media from cell supernatants or lysates. In this case, IL-1β content remained unchanged between genotypes over a 24-h period (Fig. 3J). Taken together, these data suggest that recombinant IL-1β is taken up by cells and degraded more efficiently intracellularly in MT5-MMP-deficient cells, rather than by extracellular proteinases.

### Effects of MT5-MMP deficiency and IL-1β on N-cadherin levels and processing

**Fig. 4:**
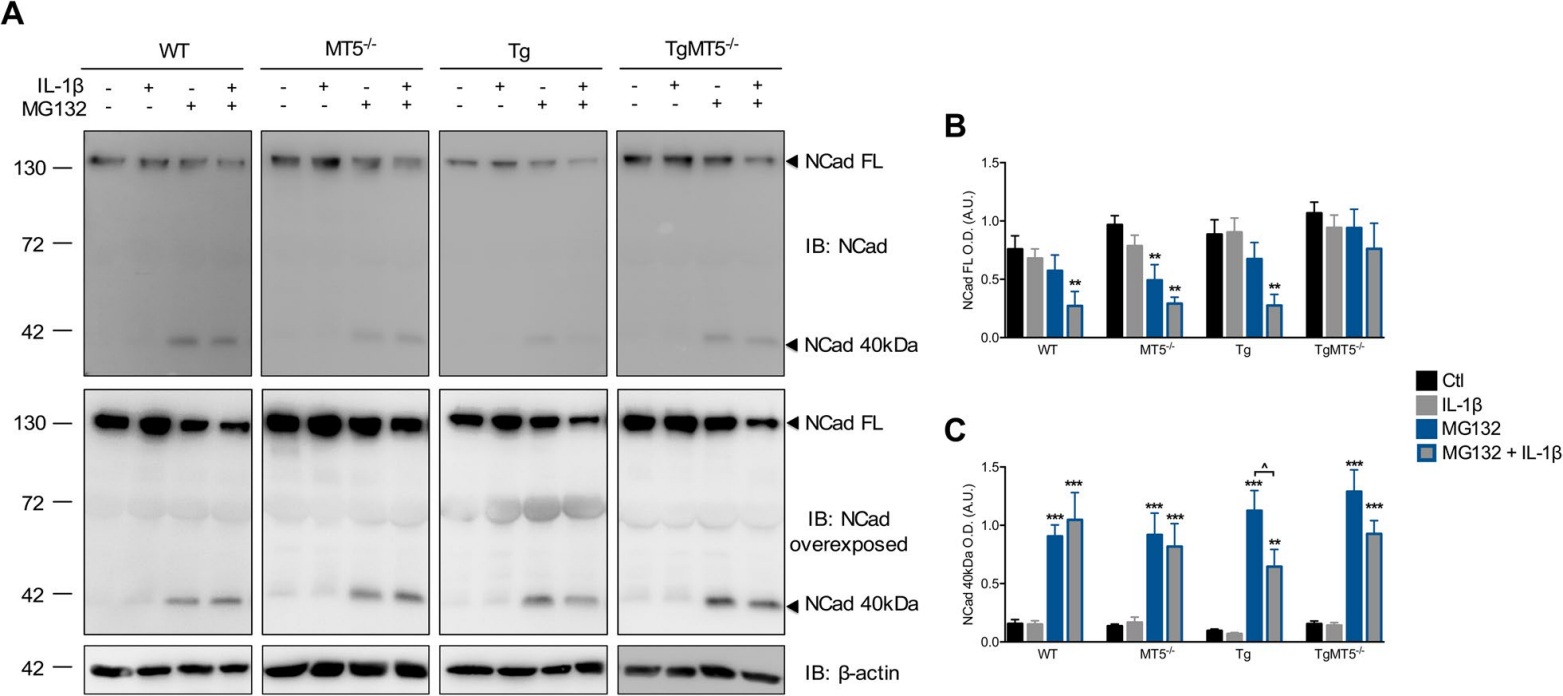
Effects of MT5-MMP deficiency and MG132 treatment on N-cadherin levels. Immunoblot analyses of full length (NCad FL) and 40 kDa N-cadherin (NCad 40 kDa) levels in control and MG132 conditions, treated or not with IL-1β, and their representative quantifications normalized with β-actin. Black bars represent control (untreated) conditions, grey bars IL-1β-treated conditions (10 ng/mL for 24 h), blue bars MG132-treated conditions (5 μM for 24 h), and grey bars with blue borders MG132 and IL-1β co-treated conditions. Values are the mean ± SEM of 4–12 independent cultures by genotype. Values are presented as % of the control ^#^*p* < 0.05 between genotypes. ANOVA followed by post hoc Fisher’s LSD test. *IB* immunoblot, *O.D.* optical density, *A.U*. arbitrary units

In view of the effects of MT5-MMP deficiency on IL-1β inflammatory response in our cultures, we asked next whether this might be related with a potential deficient processing of MT5-MMP substrate, N-cadherin. The rationale behind this question is that deficient cleavage of N-cadherin in the PNS was reported as a possible mechanism explaining the lack of inflammatory response to IL-1β injection in MT5-MMP-deficient mice [10]. For this reason, we looked for changes in the levels of canonical N-cadherin or its breakdown products resulting from MT5-MMP proteolytic activity. As shown in Fig. 4A, basal levels of full length N-cadherin (NCad FL) remained relatively stable across genotypes (Fig. 4A) and IL-1β treatment did not change this pattern (Fig. 4A and B), suggesting no significant impact of MT5-MMP deficiency on N-cadherin stability. Since N-cadherin breakdown products were not detected, we used a proteasome inhibitor MG132, previously shown to stabilize N-cadherin fragments resulting from MT5-MMP proteolysis [8]. First, we observed that inhibition of the proteasome caused a significant decrease in N-cadherin levels in WT and Tg cells treated with IL-1β and MG132, which could imply the activation of a more efficient degradation pathway, alternative to the proteasome. In MT5^−/−^ cells, such decrease was also observed after MG132 treatment alone. Only TgMT5^−/−^ cells showed unchanged levels of NCad FL in all experimental conditions (Fig. 4A and B). In agreement with a previous report, a C-terminal fragment of ~ 40 kDa was detected following MG132 treatment [8] (Fig. 4A and C), indicating proteasome degradation of this fragment in normal conditions. The level of N-cadherin 40 kDa (NCad 40 kDa) remained stable across genotypes and treatments except in Tg cells, where combined MG132 and IL-1β caused a 43% decrease compared with MG132 alone. Such decrease was prevented in TgMT5^−/−^ cells (Fig. 4A and C), possibly reflecting the relative stability of NCad FL levels in this genotype regardless of the treatment. Taken together, these data suggest that MT5-MMP is unlikely implicated in N-cadherin processing in our experimental conditions, as its deficiency does not reduce the generation of breakdown products. However, MT5-MMP absence in an inflammatory condition might interfere with the activation of alternative degradation pathways in case of impaired proteasome function.

### Effects of MT5-MMP deficiency and IL-1β treatment on baseline spontaneous synaptic activity in primary cortical neurons

**Fig. 5:**
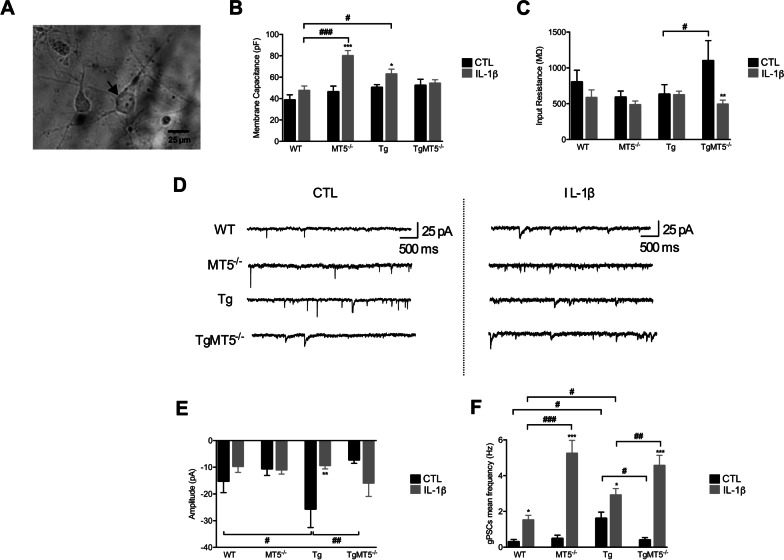
Effects of MT5-MMP on basal synaptic transmission in cortical primary neurons.** A** Microphotograph of a representative pyramidal shaped neuron (arrow) in a 11–14 DIV culture. Scale bar: 25 μm. **B** Histograms of membrane capacitance (pF; pico Farad) and **C** input resistance (MΩ: mega Ohm) of WT, MT5^−/−^, Tg, and TgMT5^−/−^ recorded pyramidal neurons. Black bars represent control (untreated) conditions (*n* = 13, 8, 11 and 10 neurons for each genotype, respectively). Grey bars represent IL-1β treatment 10 ng/mL for 24 h (*n* = 11, 10, 13, and 16 neurons for each genotype, respectively). **D** Representative traces of miniature global post-synaptic currents (gPSCs) obtained for a voltage clamped at − 50 mV in control (left) and IL-1β-treated conditions (right) (pA: pico Ampere; ms: millisecond). **E** and **F** Histograms showing the mean values of gPSCs amplitude (pA), frequency (Hz: Hertz). Black bars represent control (untreated) conditions (*n* = 12, 8, 10 and 7 neurons for each genotype, respectively). Grey bars represent IL-1β treatment 10 ng/mL for 24 h (*n* = 8, 10, 10, 12 neurons for each genotype, respectively). Values for **B**, **C** and **E**, **F** are the mean ± SEM of recorded neurons from 3 independent cultures. *p < 0.05, **p < 0.01 and ***p < 0.001 between untreated and treated cultures in the same genotype; ^#^*p* < 0.05, ^##^*p* < 0.01 and ^###^*p* < 0.001 between genotypes. ANOVA followed by post hoc Fisher’s LSD test

We and others previously reported that MT5-MMP can modulate neuronal activity [8, 9, 12, 13]. We therefore investigated how the putative functional interaction between MT5-MMP and IL-1β could affect spontaneous synaptic activity of cortical pyramidal cells at 11–14 DIV, when a functional network is already in place [42]. Figure 5A shows a representative snapshot of a recorded pyramidal neuron. In untreated control cultures, membrane capacitance (ranging from 38 to 52 pF) and input resistance (ranging from 590 to 1100 MΩ) were monitored after piercing the cell membrane in voltage-clamp mode. Membrane capacitance, which roughly represents the volume of the cell body and proximal branching, was similar across genotypes in untreated conditions. Conversely, IL-1β treatment induced significant increases of membrane capacitance by 72% and 26%, respectively, in MT5^−/−^ and in Tg cells compared with their untreated controls (Fig. 5B). In addition, IL-1β increased capacitance in MT5^−/−^ and Tg cells by 68% and 32% compared with treated WT cells, respectively. Input resistance is interpreted as a control of the neuron electric integrity, where the differences could highlight qualitative and quantitative changes in ion channels at the membrane surface. In this case, we noted that untreated TgMT5^−/−^ cells had a 74% higher input resistance than Tg (Fig. 5C). After IL-1β treatment, no differences were observed between genotypes, which averaged around 500–600 MΩ, with the notable exception of TgMT5^−/−^, where IL-1β treatment prevented the increase in cell resistance observed in untreated cells (Fig. 5C).

Figure 5D shows representative traces for each genotype in untreated (left) and treated (right) cultures. We recorded miniature global post-synaptic currents (gPSCs) in gap-free mode during 5 min, with the voltage clamped at − 50 mV. gPSCs were then analyzed off line and selected individually using the pClamp routine (Fig. 5D). The mean peak amplitude of gPSCs ranged from − 7 pA to − 25 pA, with a maximum value for Tg neurons and a minimum for the two MT5-MMP-deficient genotypes (Fig. 5E). The peak amplitude significantly increased by 69% in untreated Tg neurons compared with WT cells. Such increase was not observed in TgMT5^−/−^ neurons, which had 72% lower levels compared with Tg neurons. Likewise, the increase in peak amplitude in Tg neurons was prevented by IL-1β, which decreased levels to 64% of the value in untreated Tg neurons (Fig. 5E). In terms of basal event frequency, Tg neurons had a 536% higher value than WT neurons (Fig. 5F). Again, the increase was prevented in TgMT5^−/−^ neurons, where the levels decreased by 75% with respect to Tg. IL-1β treatment significantly exacerbated frequency in all genotypes, but the increase was surprisingly particularly important in the MT5^−/−^ and TgMT5^−/−^ groups, with > 1000% in both compared with their untreated controls (Fig. 5F). In comparison, frequency augmented by 500% in WT-treated neurons and by only 81% in Tg-treated neurons, relative to their untreated controls (Fig. 5F). Although basal frequency was already much higher in Tg cells compared to WT cells, IL-1β was still able to increase frequency in Tg cells by nearly twofold (Fig. 5F).

### Effects of MT5-MMP deficiency and IL-1β on APP metabolism

**Fig. 6:**
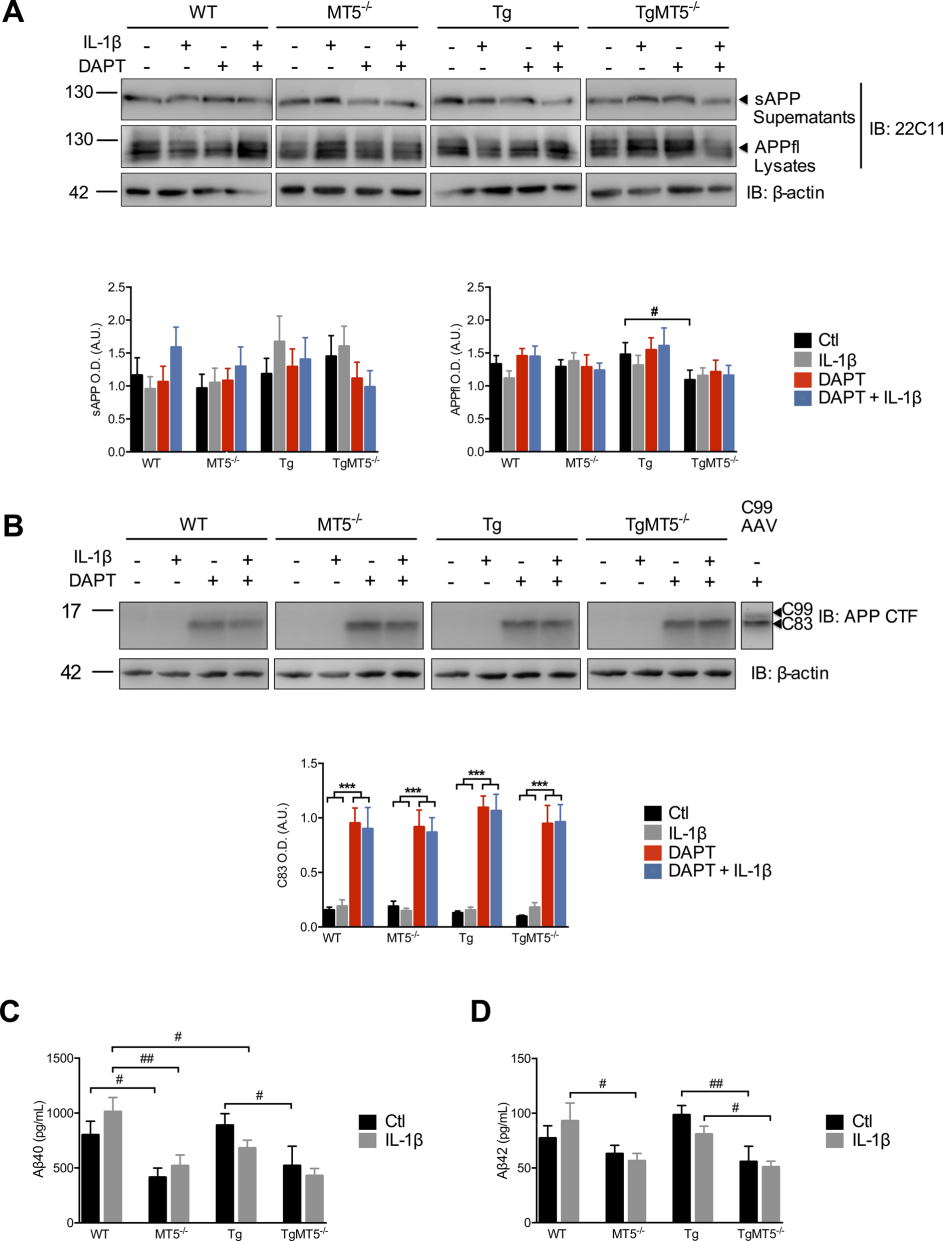
Effects of MT5-MMP deficiency and IL-1β on APP metabolism in cortical neural cell cultures.** A** Immunoblot analyses of soluble APP (sAPP) and canonical full length APP (APPfl) detected with the 22C11 antibody in primary cultures treated or not with IL-1β (10 ng/mL) and/or DAPT (10 μM), and the corresponding β-actin normalized quantifications. **B** Immunoblot analyses of APP CTF fragments detected with APP-CTF antibody in primary cultures treated or not with IL-1β (10 ng/mL) and/or DAPT (10 μM), and the corresponding β-actin normalized quantifications. AAV-C99 (right) indicates a positive control. WT cells were infected for 5 days with AAV-C99 and recovered at 11 DIV. Note that only C83 levels were detectable with DAPT treatment. **C** and **D** Measurements by MSD multiplex assay of Aβ40 and Aβ42 levels (pg/mL) in primary cultures in control (black) and IL-1β (grey) conditions. Values are the mean ± SEM of 8–16 for **A**, **B** and 4–5 for **C**, **D** independent cultures by genotype. *p < 0.05 and ***p < 0.001 between untreated and treated cultures with IL-1β and DAPT in the same genotype; ^#^*p* < 0.05, ^##^*p* < 0.01 between genotypes. ANOVA followed by post hoc Fisher’s LSD test. *IB* immunoblot, *O.D.* optical density, *A.U.* arbitrary units

Changes in inflammatory markers in AD are often associated with the accumulation of Aβ following amyloidogenic processing of APP [43, 44]. Knowing that MT5-MMP can modulate APP/Aβ metabolism [13, 17], we asked whether the apparent pro-inflammatory action of MT5-MMP might result from its ability to stimulate APP metabolism and Aβ accumulation. To address this question, we first measured the levels of secreted (sAPP) or cellular full-length APP (APPfl) using an antibody directed against the N-terminal portion of APP (i.e*.*, 22C11). This revealed no change in sAPP levels between genotypes, and only a significant decrease of 27% for APPfl in TgMT5^−/−^ cells compared with Tg (Fig. 6A). Treatments with IL-1β and/or γ-secretase inhibitor DAPT did not affect sAPP or APPfl levels (Fig. 6A). DAPT was primarily intended to block γ-secretase-mediated processing of CTFs to stabilize them and thus facilitate their detection. This was important to compare our experimental setting with in vivo work reporting brain accumulation of C99 preceding that of Aβ in 3xTg and 5xFAD mouse models of AD [34, 45], as well as the decrease of C99 and C83 upon MT5-MMP deficiency in 5xFAD mice [13, 17]. After DAPT and immunoblot with the APP CTF antibody, we detected a single band corresponding to the expected size of C83 that was not altered by MT5-MMP deficiency or IL-1β treatment (Fig. 6B). In contrast, no band corresponding to the size of C99 was detected with any of the three antibodies tested: APP-CTF (Fig. 6B), 6E10 (which recognizes human APP and its fragments containing the N-terminal of C99; data not shown) or 82E1, which recognizes the neoepitope in the N-terminal of C99/Aβ (Asp1) generated by β-secretase cleavage (data not shown). The absence of C99 was further confirmed after subcellular fractioning of membranous, cytosolic and nuclear compartments (Additional file  1). In contrast, C83 was slightly detected only at the membrane in control conditions but its levels dramatically increased upon DAPT treatment in this fraction, and interestingly, also in the nucleus, although to a lesser extent (Additional file 1). It is noteworthy that the APP-CTF and 6E10 antibodies did not detect any immunoreactive band around 30–40 kDa compatible with the expected size of the η-CTF fragments.

Next, we measured the levels of murine Aβ38, Aβ40 and Aβ42. Aβ38 was not detected in our cultures (not shown) and we found no increase of either species in Tg compared with WT cultures (Fig. 6C). However, MT5-MMP deficiency significantly reduced Aβ40 levels in MT5^−/−^ (48%) and TgMT5^−/−^ (41%) cells, compared with WT and Tg cells, respectively (Fig. 6C). Although IL-1β did not affect intragenotype Aβ40 levels, it caused a significant reduction in Tg (33%) and MT5^−/−^ cells (49%) compared with WT (Fig. 6C). Basal Aβ42 levels were approximately tenfold lower than those of Aβ40. The lack of MT5-MMP in this case reduced by 44% the levels of Aβ42 only in TgMT5^−/−^ cells compared with Tg (Fig. 6D). IL-1β had not effect on Aβ levels (Fig. 6C and D). We conclude that MT5-MMP deficiency downregulates Aβ40 and Aβ42 levels in developing neural cells in culture and that this trend is not modified by IL-1β after 24 h of incubation.

Human Aβ40 was detected only in Tg and TgMT5^−/−^ cells, albeit at relatively low concentrations (~ 35 pg/mL), as revealed by a human-specific ELISA kit (Additional file 2A), indicating that the Thy1 neuronal promoter was functional to drive human transgene expression and efficient metabolization of human APP. This is consistent with previous data showing activation of the Thy1 promoter at 4–5 DIV [46] and with the detection of *hAPP* and *hPSEN1* mRNAs in our cultures (Additional file 2B and C). Genotype or IL-1β treatment did not affect the content of hAβ40, *hAPP* or *hPSEN1* gene expression in any way in our experimental conditions (Additional file 2A–C).

### Expression of genes involved in Aβ production and degradation

**Fig. 7:**
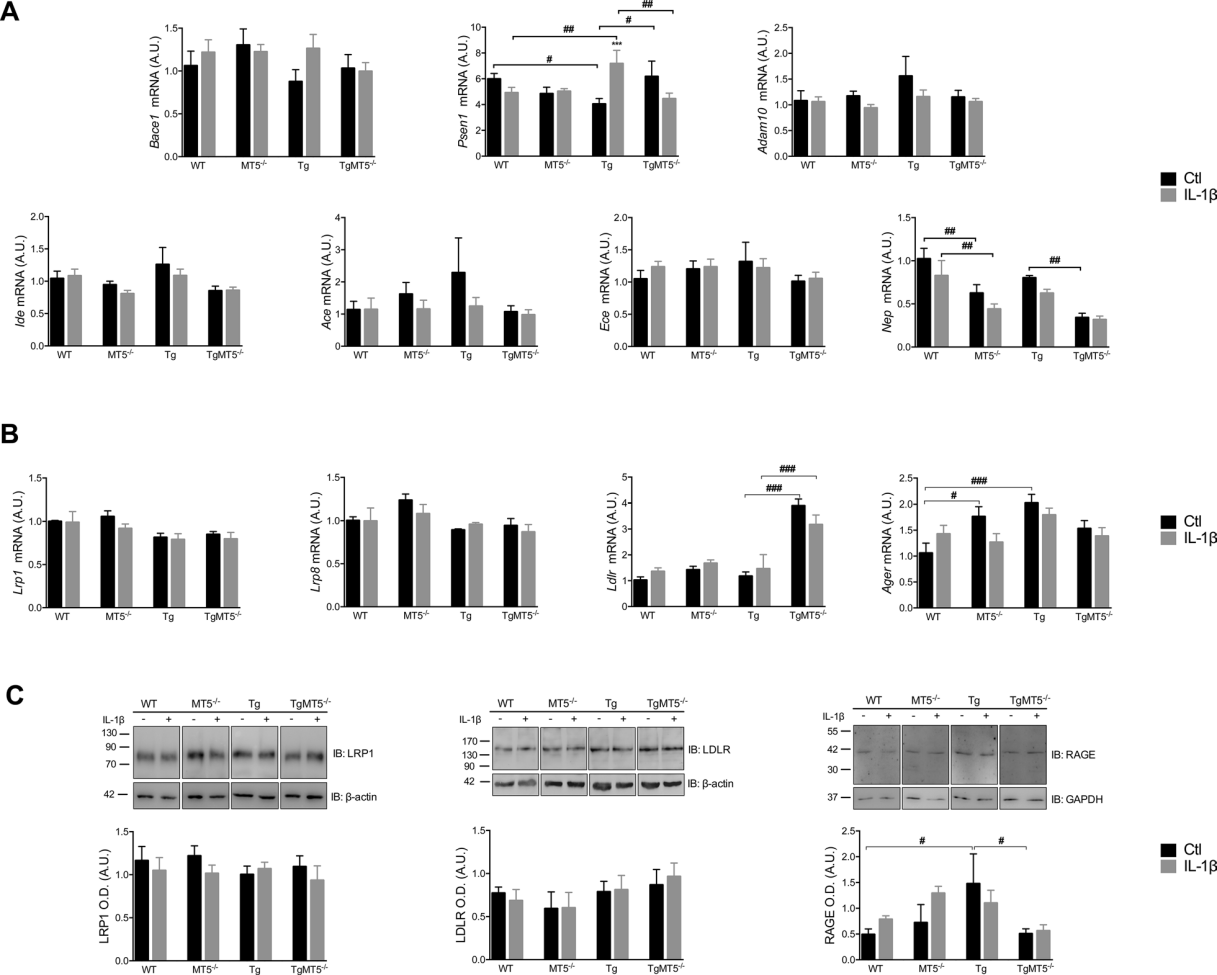
Effects of MT5-MMP deficiency and IL-1β on APP metabolism modulators in cortical neural cell cultures.** A** mRNA levels of *Bace1*, *Psen1*, *Adam10*, *hAPP*, *hPsen1*, *Ide*, *Ace, Ece1* and *Mme* measured by RT-qPCR. **B** mRNA levels of *Lrp1*, *Lrp8*, *Ldlr* and *Ager* by RT-qPCR. All RT-qPCR results are normalized with *Gapdh* as housekeeping gene. **C** Immunoblot analyses and the corresponding normalized quantifications of LRP1, LDLR with β-actin, and RAGE with GAPDH. Note that LRP8 was not detected in any cell culture. Black bars represent control (untreated) conditions and grey bars IL-1β treated conditions (10 ng/mL for 24 h). Values for A are the mean ± SEM of 6–9 independent cultures by genotype; for B 3–5 and for C 6–9 for LRP1 and LDLR and 3 for RAGE. Values are presented as % of the control. ***p < 0.001 between untreated and treated cultures with IL-1β and DAPT in the same genotype; ^#^*p* < 0.05, ^##^*p* < 0.01 and ^###^*p* < 0.001 between genotypes. ANOVA followed by post hoc Fisher’s LSD test. *IB* immunoblot, *O.D.* optical density, *A.U.* arbitrary units

Because Aβ content results from a balance between production and degradation, we assessed possible changes in the gene expression of enzymes implicated in these processes, e.g*.*, BACE1 (*Bace1*), presenilin 1 (*Psen1*), ADAM10 (*Adam10*), insulin-degrading enzyme (*Ide*), angiotensin-converting enzyme (*Ace*), endothelin-converting enzyme (*Ece*) and neprilysin (*Mme*). Only *Psen1* and *Mme* showed significant changes. In basal conditions, Tg cells expressed 33% lower levels of *Psen1* mRNA compared with WT cells and 35% lower compared with TgMT5^−/−^ cells. IL-1β induced a 79% increase of *Psen1* mRNA levels in Tg compared with untreated cells, and this increase was also significant when compared to WT (46%) and TgMT5^−/−^ cells (60%) under the same conditions (Fig. 7A). *Mme* expression was clearly downregulated in the absence of MT5-MMP. In MT5^−/−^ and TgMT5^−/−^ cells, *Mme* mRNA was down by 40% and 58% compared with untreated WT and Tg cells, respectively. IL-1β did not significantly modify the intragenotype values, but significant decreases of 47% and 49% were observed in MT5^−/−^ and TgMT5^−/−^ cells, compared with WT and Tg cells, respectively (Fig. 7A).

Cellular receptors such as low-density lipoprotein receptor-related protein 1 (LRP1) [47–50], LRP8 [51], LDLR [52] or RAGE [53] may also affect APP metabolism by either modulating the activities of β- and γ-secretase and/or directly Aβ levels through endocytosis. In this context, *Lrp1* and *Lrp8* mRNA expression was stable in all experimental groups (Fig. 7B). In contrast, *Ldlr* mRNA content was significantly upregulated by 231% in TgMT5^−/−^ compared with Tg, and IL-1β did not alter this trend. The RAGE receptor encoded by the *Ager* gene, showed no differences upon IL-1β stimulation. Nevertheless, under basal conditions, MT5^−/−^ and Tg cells expressed significantly more *Ager* than WT cells (67% and 92%, respectively) (Fig. 7B). Next, we assessed the protein content of these receptors by WB. In our experimental conditions, LRP8 was undetectable and no differences were observed between genotypes and treatment for LRP-1 and LDLR (Fig. 7C). Tg cultures expressed significantly higher levels of RAGE compared to WT (200%) and TgMT5^−/−^ (194%) (Fig. 7C). IL-1β treatment did not modulate RAGE content in all genotypes.

Overall, there was no clear evidence of transcriptional regulations that could explain the downregulation of Aβ content upon MT5-MMP deficiency. The results also indicated that incubation of 10 ng/mL of IL-1β for 24 h did not impact the expression of genes encoding potential modulators of Aβ balance, and confirmed overall no influence of the cytokine in global APP/Aβ metabolism in our experimental settings.

### Overexpression of C99 reveals the potential of MT5-MMP to control its accumulation in CNS cells

**Fig. 8:**
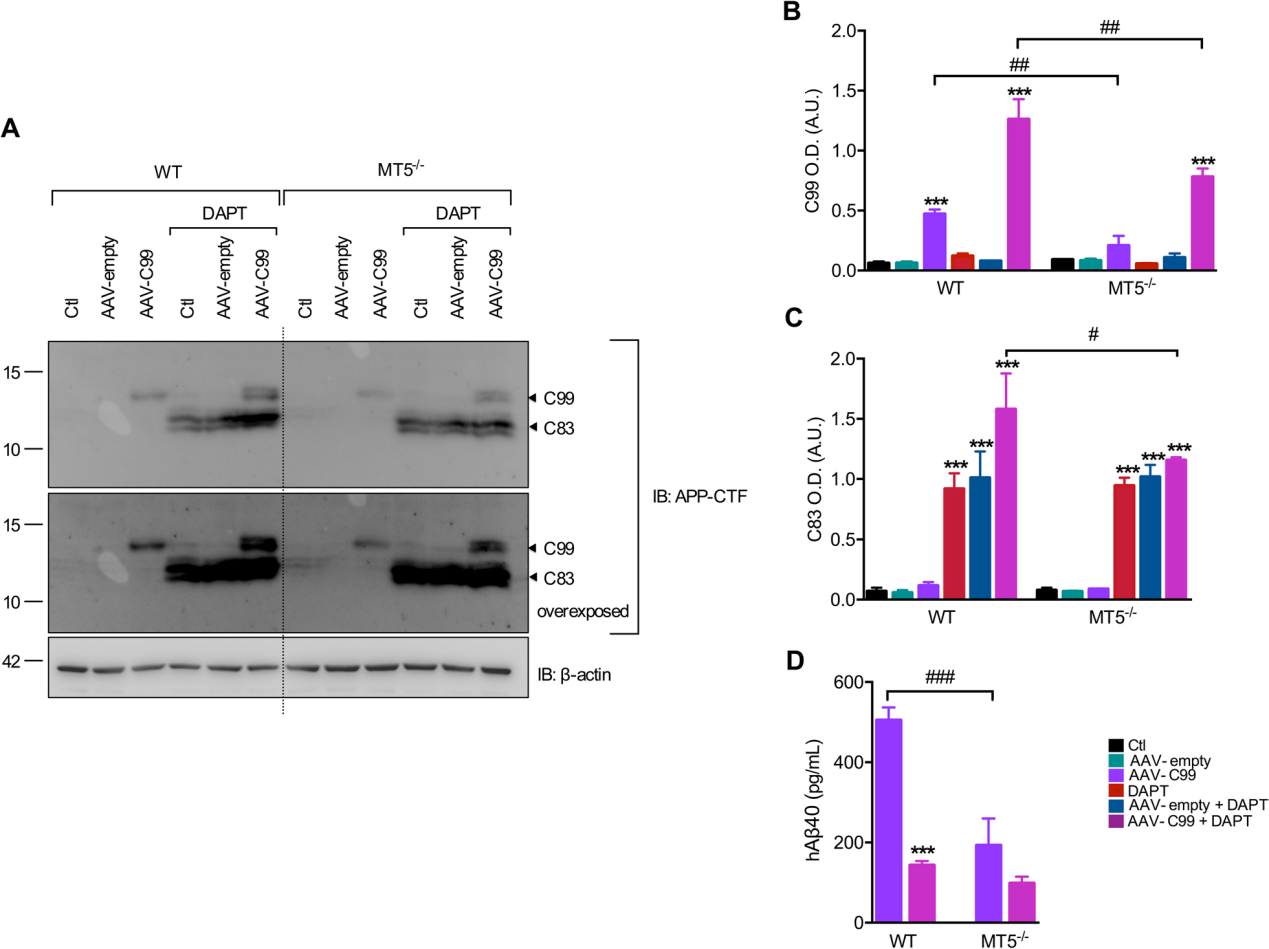
Effects of MT5-MMP deficiency on C99 overexpression in cortical neural cell cultures.** A** Immunoblot analyses of C99 and C83 detected with the APP-CTF antibody in primary cortical cultures after transduction with AAV-empty or AAV-C99 (2 μL at 5.10^12^ vg/mL), treated or not with DAPT (10 μM), and the corresponding β-actin normalized quantifications for C99 (**B**) and C83 (**C**). **D** Measurement of human Aβ40 levels (pg/mL) in primary cortical cultures using the ThermoFisher Scientific ELISA kit. Values are the mean ± SEM of 3–5 independent cultures by genotype. **p < 0.01 and ***p < 0.001 between untreated and treated cultures with DAPT in the same genotype; ^#^*p* < 0.05, ^##^*p* < 0.01 and ^###^*p* < 0.001 between genotypes. ANOVA followed by post hoc Fisher’s LSD test. *IB* immunoblot, *O.D.* optical density, *A.U.* arbitrary units

Under our experimental conditions, the incipient expression of *hAPP* and *hPSEN1* transgenes carrying AD mutations and/or acute IL-1β challenge were not sufficient to trigger the accumulation of C99 characteristic of AD, although MT5-MMP deficiency downregulated Aβ levels. These data likely reflect extreme lability and/or relatively low production of endogenous C99 in developing neurons. Taken together, this could contribute to the inability to detect steady-state C99 levels and thus mask a putative impact of MT5-MMP in C99 metabolism, as previously shown in adult 5xFAD mice [13, 17]. To circumvent this difficulty and assess whether MT5-MMP could actually regulate C99 levels in developing neurons, we overexpressed human C99 in WT and MT5^−/−^ cultures using an AAV-C99 under the control of the neuron-specific synapsin promoter [29]. As shown in Fig. 8A–C, in non-transduced cells or in cells transduced with an empty AAV, CTFs did not spontaneously accumulate and DAPT rescued only C83, but not C99 levels, consistent with C83 being a preferential substrate of γ-secretase [32] and the most abundant APP fragment in cultured neurons [15, 16]. Furthermore, MT5-MMP deficiency did not alter basal C83 after DAPT treatment (Fig. 8A and C), mirroring results in Fig. 6B. As expected, neurons transduced with AAV-C99 accumulated C99 and, most interestingly, its levels were significantly reduced by 56% in MT5^−/−^ cells compared with WT cells (Fig. 8A and B). C83 was undetectable in DAPT-free conditions (Fig. 8A and C). Conversely, DAPT treatment caused a 168% and 1300% increase in C99 and C83 in WT cells compared to untreated AAV-C99 cells, respectively (Fig. 8A–C). This high accumulation of CTFs was significantly reduced in MT5^−/−^ cells by 39% for C99 and 38% for C83. Consistent with these data, ELISA showed a 62% decrease of human Aβ40 levels in MT5^−/−^ cells transduced with AAV-C99, compared with WT (Fig. 8D). As anticipated, DAPT nearly blocked Aβ formation from C99 in WT cells (Fig. 8D), whereas the effect was negligible on MT5^−/−^ cells because their Aβ content was already very low (Fig. 8D). We conclude that MT5-MMP deficiency effectively prevents the accumulation of C99, a major pathogenic feature of AD and, more interestingly, that this can occur in developing neural cells.

## Discussion

This study provides the first experimental evidence that MT5-MMP deficiency tunes down neuroinflammation, APP metabolism and neuronal excitability in primary cortical cultures of AD and non-AD mice. This occurs as early as 11 DIV, with stable neuronal and astrocyte markers across genotypes, and upregulated levels of MT5-MMP in Tg cells. We found no clear evidence of cross-regulation between neuroinflammation and APP metabolism in young neural cells, as the effects of MT5-MMP deficiency on downregulation of IL-1β signaling and Aβ production were not cumulative. In addition, neuroinflammation caused by IL-1β treatment did not impact the levels of APP metabolites (e.g*.*, Aβ and APP CTFs), which were instead controlled by the presence or absence of MT5-MMP. Proteinase deletion also prevents spontaneous hyperexcitability in Tg neurons, but paradoxically exacerbates the frequency of events upon IL-1β treatment. Overall, MT5-MMP appears to be an enzyme capable of controlling different physiological and pathological pathways, the latter set in motion by the nascent expression of human AD transgenes in developing neural cells 11 days after seeding. This confirms the beneficial effect of MT5-MMP modulation on the early cellular dysfunctions identified, which are likely precursors to the pathogenesis of AD.

### MT5-MMP is upregulated in Tg cultures and its deficiency does not affect cell culture composition

An important finding of this work is that MT5-MMP content is higher in Tg cells, suggesting a modulating effect of AD transgenes on this proteinase. Such regulation highlights that the impact of MT5-MMP in AD, previously described in adult mice [13, 17], may actually begin at a stage well before the onset of obvious pathological and clinical signs. Of note, MT5-MMP deletion did not alter the expression of MT1-MMP, MMP-9 or MMP-2, all close MT5-MMP homologues, also involved in the control of APP metabolism and amyloidogenesis [33, 34, 54, 55], implying no compensatory regulation by these proteinases upon MT5-MMP deficiency. The content of β-III tubulin and GFAP, as well as the values of the MTT test, were similar across all experimental groups. This lack of cytotoxicity contrasts with a previous report showing cell demise in 5xFAD primary cortical neurons at 7 DIV [56]. In this study, no astrocytes were reported and cell density was fivefold lower than ours, which could explain a microenvironment that facilitates neuronal vulnerability. In addition, the apparent lack of toxicity mediated by IL-1β compared to previous studies [57, 58] may be related to different experimental settings used, including concentrations and time of exposure to the cytokine.

### MT5-MMP deficiency attenuates basal neuroinflammation and the neuroinflammatory response to IL-1β in CNS neural cells

We previously found increased levels of IL-1β in the brain of 3-day-old 5xFAD pups [59] prior Aβ accumulation, and later at 2 months, along with the onset of Aβ accumulation [34]. Interestingly, MT5-MMP deletion resulted in decreased IL-1β levels in the brains of 5xFAD mice at prodromal stages of the pathology, indicating functional interactions between IL-1β and MT5-MMP [13]. Consistent with this idea, we show here that basal IL-1β levels are higher and those of Aß are stable in Tg cells compared to WT, arguing for regulation of inflammation in young cells by a non-Aβ related mechanism. Moreover, MT5-MMP deficiency reduces the neuroinflammatory response to IL-1β as well as the basal levels of IL-1β and MCP-1. The effect of genotype/IL-1β was cytokine-selective, as shown by the lack of effect on *Tnfa*, and a more complex behavior of IL-6, whose reduction in Tg cells after IL-1β was recovered in TgMT5^−/−^. Downregulation of MCP-1 in MT5-MMP-deficient cells could dampen the system's ability to recruit microglia/macrophages to the site of inflammation, thereby helping to limit the progression of an exacerbated inflammatory response. Overall, these data echo a previous study showing that systemic injection of IL-1β did not trigger an inflammatory response in the PNS of adult MT5-MMP-deficient mice [10]. In that case, MT5-MMP-deficient prevented proper N-cadherin processing eventually disrupting the crosstalk between sensory neurons and mast cells [10]. Unchanged levels of N-cadherin or its proteolytic fragments in our MT5-MMP-deficient cells argue against this possibility. Alternatively, our data imply instead that cells lacking MT5-MMP could degrade IL-1β in a more efficient manner. This idea is indirectly supported by recent data showing that non-catalytic interactions of MT5-MMP promote C99 degradation by the proteasome and, to a lesser extent, by lysosomes [32]. Although IL-1β clearance is not well understood, it has been suggested that low levels of IL-1β stimulate autophagolysosomal function and attenuate inflammation in cell cultures, while higher cytokine concentrations (> 200 pg/mL) have the opposite effect [60, 61]. Whether MT5-MMP may act as an interactor/chaperon for IL-1β and/or the IL-1β/IL-1R1 complex, as it is the case for APP [13, 17, 32], needs further investigation.

### Changes in spontaneous synaptic activity depend on genotype and IL-1β treatment

To investigate the impact of MT5-MMP deficiency on basal synaptic activity, we measured spontaneous network synaptic events as a landmark for each genotype. In agreement with previous reports (see for review [62]), our Tg (5xFAD) neurons showed increased synaptic activity, as illustrated by higher amplitude and frequency, which is considered as a sign of hyperexcitability. At 11–14 DIV, with WT and Tg cells showing equal levels of Aβ (see Fig. 6), it is unlikely that the peptide influences the hyperexcitability observed in Tg neurons. The latter showed increased levels of MT5-MMP and IL-1β compared with WT, and more interestingly TgMT5^−/−^ neurons do not show hyperexcitability and show control values of IL-1β levels. Together, this raises the possibility of a coordinated action of MT5-MMP and IL-1β in promoting neuronal hyperexcitability. Although, IL-1β has diverse and sometimes divergent effects on neuronal activity depending on cell-type, cytokine concentration and duration of the stimulus [63], several studies highlight various mechanisms of IL-1β-mediated excitability: NMDA receptor stimulation of Ca^2+^ influx [64], inhibition of GABA-evoked currents [65] or prevention of the inhibitory effect of cannabinoid CB1 receptor on glutamate release [66]. In this context, it is possible that the onset of neuronal hyperexcitability observed in 5xFAD mice [67, 68] takes place during development, in which case, lower levels of IL-1β in MT5-MMP-deficient neurons could help to attenuate this process in the long run.

IL-1β elicited cell responses such as preventing an increase in amplitude in Tg cells, which could be interpreted as a homeostatic cellular response to the inflammatory burst. A possible post-synaptic mechanism of IL-1β underlying such effect could be the cytokine-mediated decrease in the content and phosphorylation of the AMPA-GluR1 subunit at the post-synaptic membrane [69]. IL-1β may also act as pre-synaptic neuromodulator [70, 71], which is consistent with the increased frequency we observed in all genotypes. However, the magnitude of the effect of MT5-MMP deficiency is surprising, given the weak inflammatory response of MT5^−/−^ cells to IL-1β (see Fig. 3), raising the possibility that MT5-MMP could differentially affect various IL-1β signaling pathways mediated [63] or not [72] by membrane receptors. Furthermore, as the increase in capacitance correlates with the increase in membrane surface area and pre-synaptic vesicle fusion [69], a parallelism could be established with the observation of increased gPSCs and capacitance in IL-1β-treated MT5^−/−^ neurons. However, no general conclusion can be drawn, as this correlation was not validated in the other experimental groups. Alternatively, the lack of MT5-MMP could prevent the formation of sAPP95/sAPPη, recently suggested to bind the GABA_B_R1a and inhibit pre-synaptic neurotransmitter release [73]. Even if we found no sAPP around 85–95 kDa, we cannot exclude that a small functional pool of this WB-undetectable fragment reaches the synapse. Although further research is needed to better understand the novel effects reported here, MT5-MMP deficiency appears to prevent AD genotype-related synaptic dysfunction under basal conditions, while exacerbating IL-1β-induced neuronal excitability.

### MT5-MMP deficiency reduces Aβ levels and has no effect on endogenous CTFs

In contrast to previous observations in the brains of adult 5xFAD mice [13, 17, 34], C99 did not accumulate in developing neural cells. Yet, murine Aβ implicitly proved the formation at some point of its immediate precursor, C99. We assume that the latter is formed at a low pace and/or that it is promptly degraded by the proteasome or autophagolysosome [32], and even by α-secretase to yield C83 [32, 74]. Likely, all or some of these mechanisms are active in developing neural cells, thus preventing the neurotoxic effects of C99 accumulation [29, 45, 75]. In contrast to C99, DAPT did rescue C83 levels, which were not altered by MT5-MMP deficiency. This is in agreement with data showing stable levels of C83 in the frontal cortex of 5xFAD mice lacking MT5-MMP [17]. Unlike CTF modulation, MT5-MMP deficiency resulted in a decrease of Aβ levels, which could not be correlated with increases in Aβ-degrading enzymes. In fact, the observed reduction in neprilysin *(Mme)* is somewhat counterintuitive, unless it actually underlies a negative feedback response to a potential increase in neprilysin activity. Analysis of genes involved in Aβ transport/clearance (e.g*., Lrp1*, *Lrp8*, *Ldlr*, *Ager*) also revealed no clear evidence of a transcriptional mechanism that could explain the modulation of Aβ content.

### MT5-MMP deficiency prevents the accumulation of overexpressed C99 and hence Aβ

The above data denote the capacity of MT5-MMP to control Aβ levels, echoing our pioneer work in vivo [13], and demonstrate that functional interactions of MT5-MMP with APP/Aβ may already happen in developing neural cells. However, the inability to detect C99 in these cells led us to question whether MT5-MMP might also control C99 as it does in adult mice [13, 17]. We addressed  this  question by providing evidence that the levels of transduced human C99 were downregulated in MT5-MMP-deficient primary neurons and, therefore, also those of Aβ. It is noteworthy that C83 derived from overexpressed C99 was also downregulated in MT5-MMP-deficient neurons, in contrast to the lack of effect on constitutive C83 likely resulting from APP processing. Thus, the control of C83 by MT5-MMP seems to depend mostly on whether it is generated from APP or C99. Taken together, these data indicate that young neurons have the potential to prevent the accumulation of endogenous C99 and thus prevent the derived detrimental consequences. This work  also highlights that MT5-MMP deficiency facilitates C99 degradation in these neurons, which supports our recent results in HEK cells showing that deletion of C-terminal domains of MT5-MMP does indeed lead to C99 degradation [32].

## Concluding remarks

The present work unveils regulatory events in developing neural cells that may influence early AD pathogenesis through functional interactions between MT5-MMP and IL-1β. It is noteworthy that inflammation and neuronal activity are particularly regulated by AD genotype and MT5-MMP in young cells, suggesting that they are important early markers of pathology onset in AD settings. Similarly, IL-1β appears to be a selective modulator of neuroinflammation and neuronal activity in these young cells, although the complexity of the effects (or lack thereof) of the cytokine must consider the limitations of our experimental setting, including for example the use of a single concentration of IL-1ß. Overall, MT5-MMP appears to be a multifaceted modulator at the crossroads of neuroinflammation, APP metabolism, and synaptic function, further enhancing interest in this proteinase and the possible therapeutic implications of its modulation in AD.

## Supplementary Information

**Additional file 1. **Immunoblots representing subcellular distribution of C83 detected with the APP-CTF antibody in primary cortical cultures at 11 DIV. Fractions are represented with their loading controls: for fraction 1, cytosolic—GAPDH; for fraction 2, membranous—Na + /K + ATPase, and for fraction 3, nuclear—Histone 3. Cells were treated or not with DAPT (10 μM). AAV-C99 (right) indicates a positive control. WT cells were infected for 5 days with AAV-C99 and recovered at 11 DIV. Note that only C83 levels were detectable with DAPT treatment.

**Additional file 2. **A Measurement of human A levels (pg/mL) in Tg and TgMT5-/- cultures using the ThermoFisher Scientific ELISA kit. B and C. mRNA levels of hAPP and hPSEN1 analyzed by RT-qPCR in Tg and TgMT5-/- cultures and normalized with Gapdh as housekeeping gene. Black bars represent control (untreated) conditions and grey bars IL-1β treated conditions (10 ng/mL for 24 h). Values are the mean ± SEM of 3–5 independent cultures by genotype.

## Data Availability

All data generated or analyzed during this study are included in this published article and are available from the corresponding author on reasonable request.

## Abbreviations

3xTG: Transgenic mice expressing human *APP MAPT* and *PSEN1* genes with 3 familial mutations
5xFAD: Transgenic mice expressing human *APP* and *PSEN1* genes with 5 familial mutations
AAV: Adeno-associated virus
A.U.: Arbitrary units
AD: Alzheimer’s disease
Aβ: Amyloid β peptide
ACE: Angiotensin converting enzyme
ADAM10: A Disintegrin And Metalloproteinase 10
APP: Amyloid precursor protein
BACE1: Beta-site APP cleaving enzyme 1 (β-secretase)
C99/C83: APP CTF of 99/83 amino acids
CTF: C-terminal fragment
DAPT: N-[N-(3,5-difluorophenacetyl)-l-alanyl]-S-phenylglycine t-butyl ester, γ-Secretase inhibitor
DIV: Days in vitro
FL: Full length
ICC: Immunocytochemistry
ECE: Endothelin converting enzyme
IB: Immunoblot
IDE: Insulin degrading enzyme
IL-1β/6: Interleukin-1β/6
LDLR: Low-density lipoprotein receptor
LRP1/8: Low-density lipoprotein related-protein 1/8
LTP: Long-term potentiation
MCP-1: Monocyte chemoattractant protein-1 (Ccl2)
MG132: Proteasome inhibitor
MMP-2/-9: Matrix metalloproteinase
MT1/5-MMP: Membrane-type 1/5-matrix metalloproteinase
O.D.: Optical density
PSEN1: Presenilin 1
RAGE: Receptor for advanced aged products
RT-qPCR: Reverse transcription-quantitative PCR
sAPP95: Soluble amyloid protein precursor fragment generated by MT5-MMP
sAPPα/β: Soluble APPα/β
sAPPFL: Full length soluble amyloid protein precursor
TTX: Tetrodotoxin
WB: Western blot
WT: Wild type
