# Supplementary figures and images for: MT5-MMP promotes neuroinflammation, neuronal excitability and Aβ production in primary neuron/astrocyte cultures from the 5xFAD mouse model of Alzheimer’s disease
Source: J Neuroinflammation. 2022 Mar 11;19:65. doi: 10.1186/s12974-022-02407-z (PMC8915472; doi:10.1186/s12974-022-02407-z)

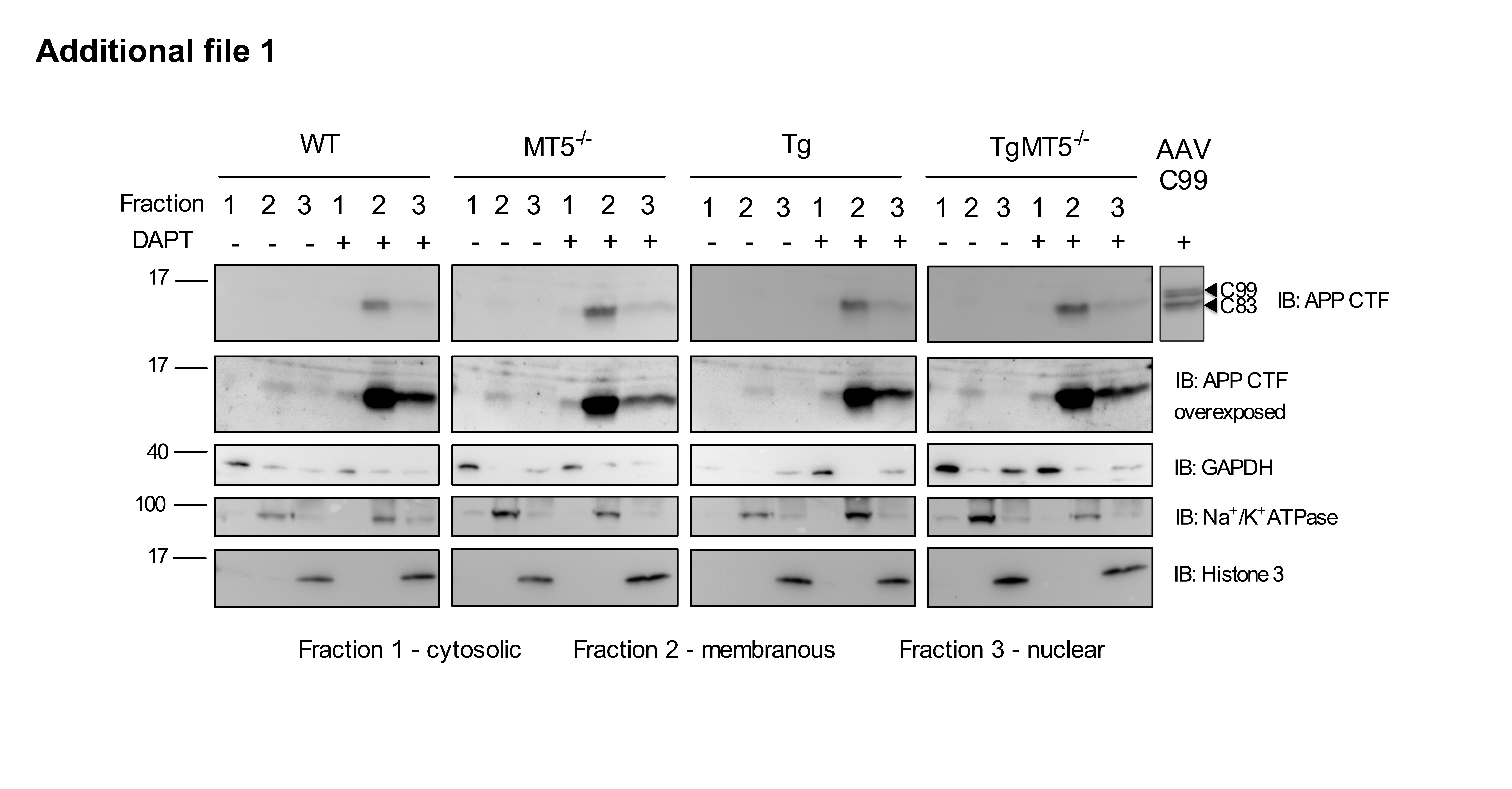

Supplement: Supplementary file 1 — Additional file 1. Immunoblots representing subcellular distribution of C83 detected with the APP-CTF antibody in primary cortical cultures at 11 DIV. Fractions are represented with their loading controls: for fraction 1, cytosolic—GAPDH; for fraction 2, membranous—Na + /K + ATPase, and for fraction 3, nuclear—Histone 3. Cells were treated or not with DAPT (10 μM). AAV-C99 (right) indicates a positive control. WT cells were infected for 5 days with AAV-C99 and recovered at 11 DIV. Note that only C83 levels were detectable with DAPT treatment. [file 12974_2022_2407_MOESM1_ESM.tif]

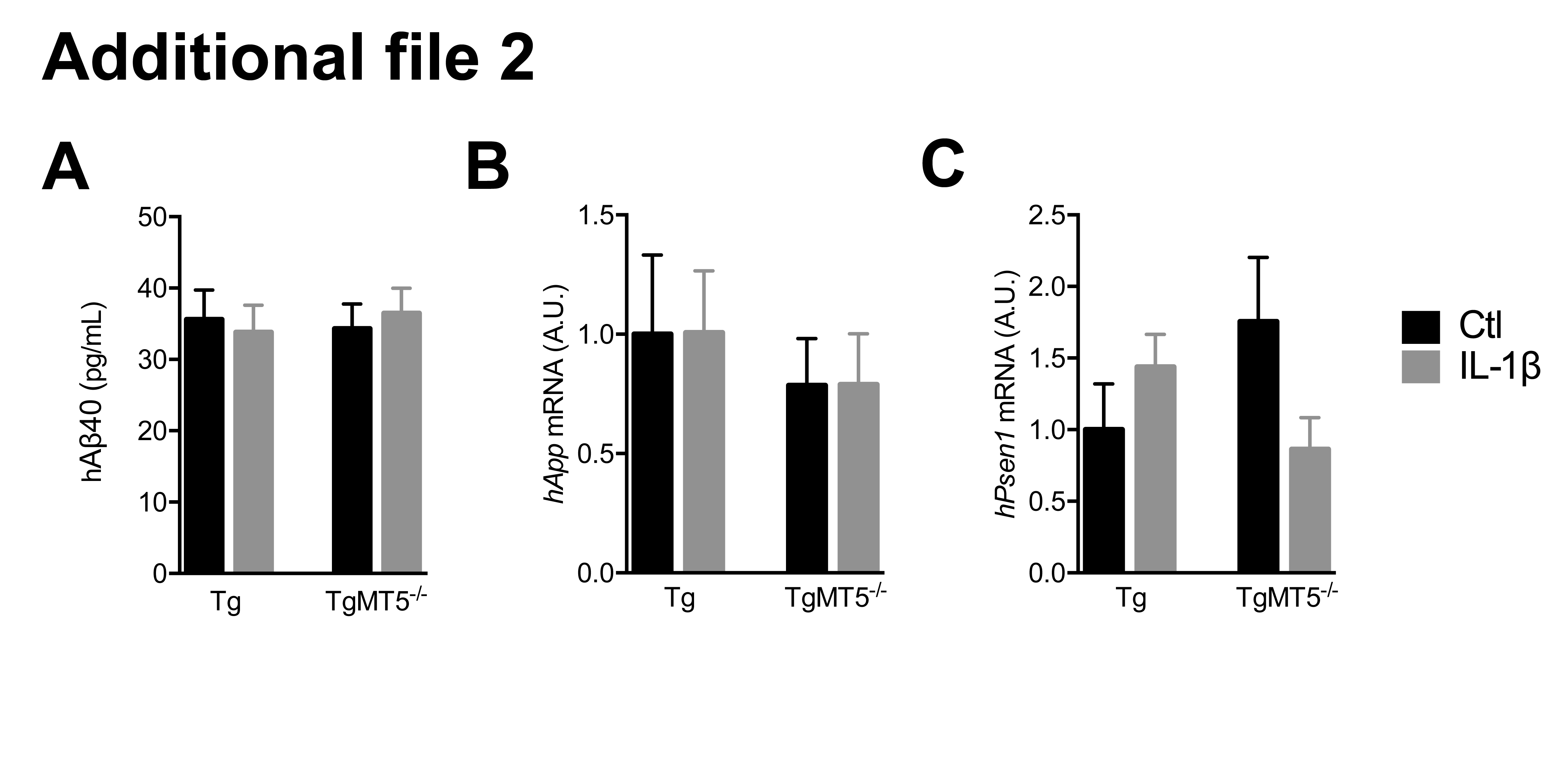

Supplement: Supplementary file 2 — Additional file 2. A Measurement of human A levels (pg/mL) in Tg and TgMT5-/- cultures using the ThermoFisher Scientific ELISA kit. B and C. mRNA levels of hAPP and hPSEN1 analyzed by RT-qPCR in Tg and TgMT5-/- cultures and normalized with Gapdh as housekeeping gene. Black bars represent control (untreated) conditions and grey bars IL-1β treated conditions (10 ng/mL for 24 h). Values are the mean ± SEM of 3–5 independent cultures by genotype. [file 12974_2022_2407_MOESM2_ESM.tif]
